# Perceptual ripening of oranges

**DOI:** 10.1177/20416695241258748

**Published:** 2024-08-03

**Authors:** Karl R. Gegenfurtner

**Affiliations:** Department of Psychology, 9175 Giessen University, Giessen, Germany

**Keywords:** color, illusions, applied perception, assimilation

## Abstract

We present a practical example for the phenomenon of color assimilation. We describe the advances in research on color assimilation from von Bezold, to Albers and Munker, and provide a compelling example of the recently described “Confetti-illusion” by Novick. Our research introduces a novel aspect by showing how unripe and greenish looking oranges can be perceived as ripe and vibrantly colored when viewed through an orange net. These findings highlight the significant implications of color assimilation in everyday consumer environments, offering a fresh perspective on how visual perception can be manipulated.

Color assimilation is a well-established phenomenon. A particularly impressive demonstration of assimilation was recently given by Novick and Kitaoka (2021) with the “Confetti illusion.” In this illusion, a neutrally colored ball shape is placed among an array of differently colored lines. When the lines of a particular color are in the foreground, the color of the ball assimilates the lines. Figure 1 presents a version, where I replaced the confetti with the faces of the three founders of trichromatic color theory. Young (1802) postulated that three types of visual receptors might do, which was later established empirically by Helmholtz (1852, 1860) and Maxwell (1857, 1860). The illusion shown in Figure 1 might be particularly strong for two reasons. First, when fixating one face, assimilation might be even stronger for the faces that are peripherally viewed, due to the decrease in spatial resolution, which is especially steep for the chromatic channels (Mullen & Kingdom, 1996; Hansen, Pracejus & Gegenfurtner, 2009). Second, we might be particularly sensitive to deviations from a normal face color, because even small deviations in facial skin coloring can signal emotion or state of health (Hasantash et al., 2019).

**Figure 1.**
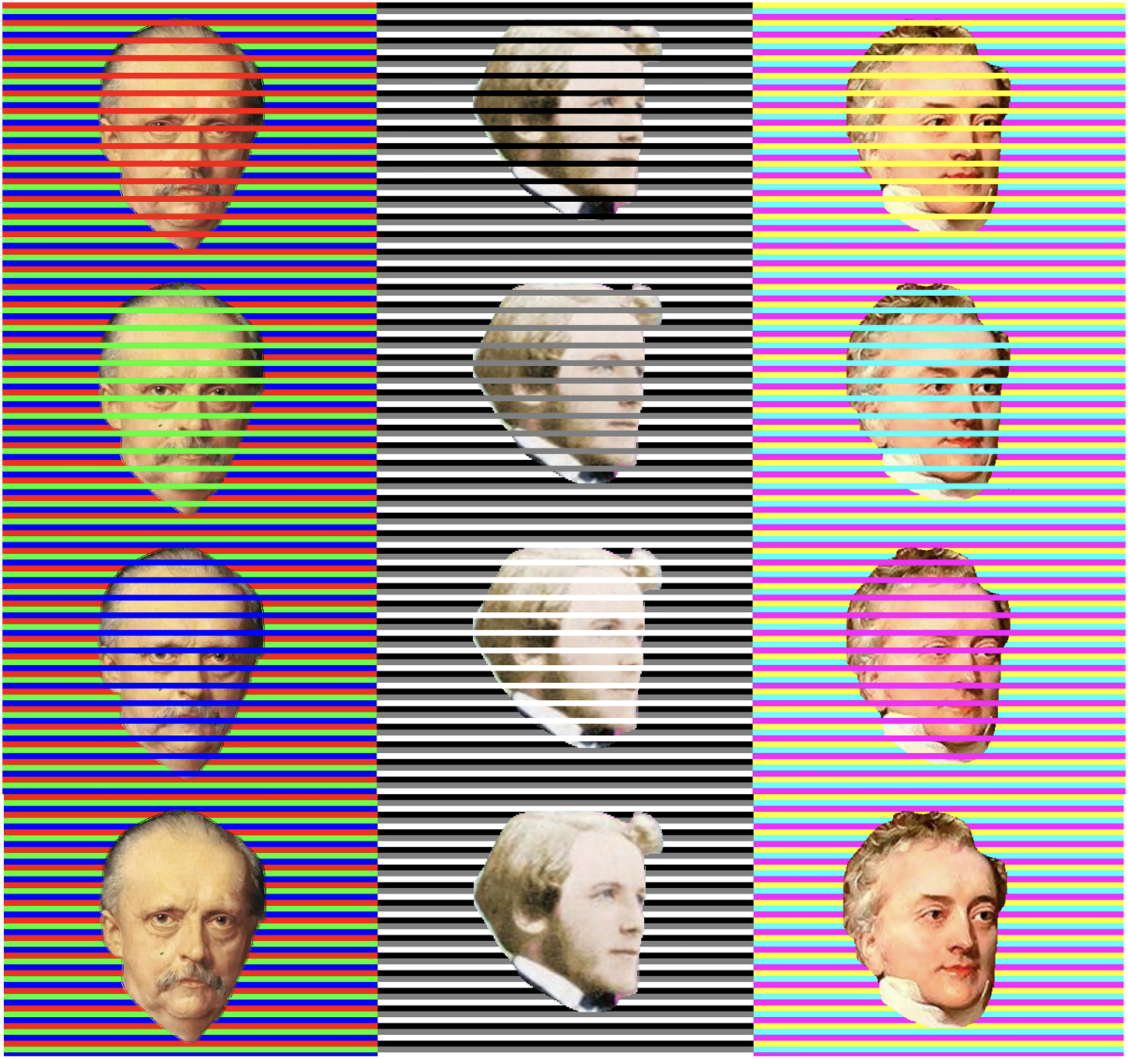
Version of the confetti illusion with the faces of Helmholtz, Maxwell and Young.

The known origins of color assimilation date back to Bezold (1874), who juxtaposed the (back then) well-known effects of color contrast with the new phenomenon later called “color spreading.”^1^ In Bezold's illustrations, the effects on color appearance are clearly visible, but still rather modest. Almost 100 years later, the artist Albers (1963) published several color assimilation demonstrations in his book “The interaction of color.” These were noticeably stronger than the earlier ones by Bezold, and they are of artistic nature. Albers named these phenomena “optical mixture,” presumably because he thought—along with the lines of von Bezold—that the phenomenon was caused by purely optical factors. Historical accounts of the developments in color assimilation were given by Jameson & Hurvich (1975), Fach & Sharpe (1986), White (2010), and Novick & Kitaoka (2021).

Nowadays, color assimilation phenomena are often named after Munker, who produced particularly compelling versions with stunning effects. Unfortunately, Munker's work was published in German language only (Schober & Munker, 1967), and the best illustrations are only included in his unpublished Habilitationsschrift^2^ (Munker, 1970). A few years after Munker, White independently discovered the grayscale version of the effect, which is since known as the White-illusion (White, 1979, for a brief history see White, 2010). Further stunning demos and thorough experimental investigations of color assimilation were done by Shevell and his colleagues (e.g., Monnier & Shevell, 2003).

Now, while the above is nice to look at, what does it have to do with oranges? Well, I buy juice oranges from my favorite fruit seller Helga every Saturday at the local market. On a recent day, she did not have any nice ripe oranges, as could be expected during the German summer. Later, stopping by in a supermarket, there seemed to be an abundance of ripe oranges, and of course I bought a bunch of them, handily packaged into an orange net for carrying.

At home, when I took out the oranges from the net, a miracle happened: each orange, beautifully colored within the net, turned into an abysmal shade of green! This can be seen in the photographs in Figure 2, where the effect of the netting is illustrated.

**Figure 2.**
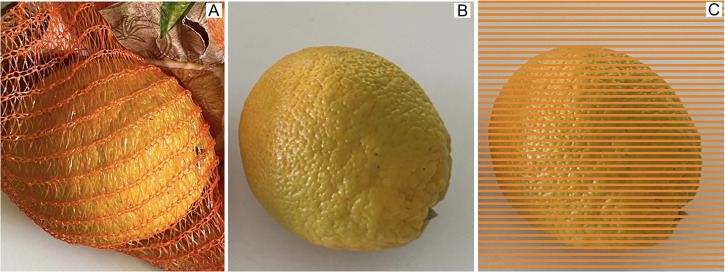
Unripe green orange (A) within the orange net, (B) in isolated view, and (C) with a “Munker-net.”

It is easy to relate the assimilation demo above with the perceptual effects here. The thin weave of the orange net is assimilated into the skin color of the orange, which then assumes its beautiful color. The effect is certainly partly due and strengthened by interreflections between the net and the fruit. Light is being reflected back and forth between netting and orange skin, and this has the tendency to increase saturation (e.g., Yu et al., 2019). Under some circumstances such interreflections can be correctly taken into account, and their effect discounted for proper color appearance (Bloj et al., 1999). In the case at hand, the memory color of the orange will also influence the perceptual interpretation (Hansen et al., 2006) and work against an interpretation of the interreflections. We have also shown recently that seeing a collection of similarly colored objects increases the perceived saturation of the individual objects (Toscani et al., 2023). The net exactly achieves this effect, as compared to an isolated orange.

In Figure 2C, we present a version of the greenish orange with an artificial Munker-style net. In this case, there are no interreflections, nor is there any surround effect. The potential effect of memory color is identical in 2B and 2C. We conclude that color assimilation alone provides a strong effect on color appearance, turning the greenish looking orange into a beautifully orange one. Needless to say that fruit vendors have realized this a long time ago. Looking around in a typical supermarkets, it is easy to see that fruits and vegetables (e.g., lemons, onions, zucchini, or even potatoes) are typically packaged in nets that are of the color of perfect exemplars.

A big chuckle for the color scientist, a sad moment for the consumer!

## Notes

1. An English translation of Bezolds book including the colored plates is available online at https://books.google.de/books?id=_vgYAAAAYAAJ&pg=PT17#v=onepage&q&f=false.

2. Habilitation is a German degree required to apply for professorships and for university teaching. It is no longer strictly necessary, but was a must back then. The corresponding theses were typically written in German language and therefore not widely read internationally.
